# α‐Fetoprotein fragment synergizes with sorafenib to inhibit hepatoma cell growth and migration and promote the apoptosis

**DOI:** 10.1111/jcmm.17565

**Published:** 2022-09-30

**Authors:** Qiujiao Wang, Wei Li, Minni Zhang, Zijuan Zou, Xu Dong, Yi Chen, Junnv Xu, Mingyue Zhu, Mengsen Li, Bo Lin

**Affiliations:** ^1^ Hainan Provincial Key Laboratory of Carcinogenesis and Intervention Hainan Medical College Haikou China; ^2^ Department of Medical Oncology, Second Affiliated Hospital Hainan Medical College Haikou China; ^3^ Institution of Tumor Hainan Medical College Haikou China

**Keywords:** AFP inhibiting fragments, drug delivery, HCC therapy, protein expression, sorafenib

## Abstract

Alpha fetoprotein (AFP) is associated with hepatocellular carcinoma (HCC) by stimulating the proliferation, metastasis and drug resistance. The application of AFP fragments to inhibit the malignant behaviours induced by AFP is a new strategy for the treatment of HCC. In an effort to design, screen and discover drugs, we attempted to express different human AFP fragments (AFP^220–609^, AFP^390–609^ and AFP^460–609^) in a Bac‐to‐Bac system. We found that the AFP^390–609^ fragment was highly expressed in the system. Then, we assessed the bioactivity of the fragment in the human liver cancer cell line Bel7402, and the results indicated that the AFP fragment synergized with sorafenib to inhibit the hepatoma cell growth and migration and promote the apoptosis. This study provides a method to produce significant AFP fragments to screen AFP inhibitors for use in HCC therapy.

## 1 INTRODUCTION

Human alpha fetoprotein (AFP) has a molecular mass of 70 kDa, which consists of 609 amino acids with glycosylation sites that may link different structures of carbohydrate moieties in different tissues and diseases.^1^ AFP contains three domains, each domain is composed of approximately 200 amino acids, and the domain3 is considered to play an important role in binding to signalling proteins and implementing the function.^2^ AFP may interact with multiple signalling molecules, including membrane receptors, PTEN, caspase‐3 and retinoid acid receptor‐β (RAR‐β), to mediate HCC cell proliferation, metastasis and apoptosis in oncogenic growth processes.^3^, ^4^ The use of AFP fragments (derived from domain3) to prevent AFP from binding to its receptors or signal transduction molecules can inhibit the malignant behaviours mediated by AFP. Additionally, the use of AFP fragments that synergize with drugs can selectively destroy cancer cells.^4^, ^5^

Alpha fetoprotein fragments not only deliver drugs that selectively destroy cancer cells which highly express AFP receptors (AFPRs), but also inhibit the role of AFP in promoting malignant behaviours of cancer cells. For example, AFP‐3BC (an AFP fragment derived domain3), which does not bind to non‐stimulated lymphocytes, can be loaded with drugs to selectively accumulate in ovarian adenocarcinoma SKOV3 cells and human breast MCF7 cells, suppressing the proliferation of these cancer cells.^6^ Other fragments, rAFP3D and r3dAFP, which are also derived from AFP domain3, can deliver drugs to cancer cells with high AFPR expression. These AFP fragments can be combined with drugs to strengthen the therapeutic effect.^7^, ^8^ These findings indicate that AFP fragments can be a promising new vector for delivering drugs or synergizing with drugs in selectively targeting and killing cancer cells.

The study was the first report of that different human AFP fragments (AFP^220–609^, AFP^390–609^ and AFP^460–609^) were expressed in a Bac‐to‐Bac system and the AFP^390–609^ fragment was the most highly expressed in the system. We also detected the bioactivity of the AFP^390–609^ fragment in the human liver cancer cell line Bel7402, which highly expresses AFPR.^9^ The results indicated that the AFP fragment synergized with sorafenib to inhibit HCC cell growth and migration and promote the apoptosis. The present study established a method for expressing and producing AFP fragments, which could be used to deliver drugs or synergize with drugs in the therapeutics of HCC.

## 2 MATERIALS AND METHODS

### 2.1 Expression vector construction

The four vectors constructed are shown in Table 1.

**TABLE 1:** Constructed vectors

Vector name	Protein sequence
pFastBac‐Dual‐AFP	Full‐length AFP including residues 1–609 with 6×His at the C‐terminus
pFastBac‐Dual‐AFP^220–609^	AFP fragment including residues 220–609 with 6×His at the C‐terminus
pFastBac‐Dual‐AFP^390–609^	AFP domain3 fragment including residues 390–609 with 6×His at the C‐terminus
pFastBac‐Dual‐AFP^460–609^	AFP domain3 fragment including residues 460–609 with 6×His at the C‐terminus

AFP (full‐length AFP including residues 1–609) gene clone: AFP (NCBI: NM001134) cDNA (from base 45 to base 1872) was synthesized with a 6×His tag sequence at the C‐terminus. Synthetic cDNA was amplified by PCR using the following primer pair: 5′‐CCGAAGCTTATGAAGTGGGTGGAATCAATTTTTT‐3′ and 5′‐CGGGGATCCTCAGTGATGGTGATGGTGATGAACTCCCAAAG‐3′.

AFP^220–609^ (AFP fragment including residues 220–609 with 6×His) gene clone: AFP (NCBI: NM001134) cDNA (from base 705 to base 1872) was synthesized with ATG at the N‐terminus and a 6×His tag sequence at the C‐terminus. Synthetic cDNA was amplified by PCR using the following primer pair: 5′‐CCGAAGCTTATG CAACAT GCATGTGCAG TAATGAAAAATTTT‐3′ and 5′‐CGGGGATCCTCAGTGATGGTGATGGTGATGAACTCCCAAAG‐3′.

AFP^390–609^ (AFP domain‐3 fragment including residues 390‑609 with 6×His) gene clone: AFP (NCBI: NM001134) cDNA (from base 1215 to base 1872) was synthesized with ATG at the N‐terminus and a 6×His tag sequence at the C‐terminus. Synthetic cDNA was amplified by PCR using the following primer pair:5′‐CCGAAGCTTATGCTTGAATGCCAAGAT‐3′ and 5′‐CGGGGATCCTCAGTGATGGTGATGGTGATGAACTCCCAAAG‐3′.

AFP^460–609^ (AFP domain‐3 fragment including residues 460–609 with 6×His) gene clone: AFP (NCBI: NM001134) cDNA (from base 1425 to base 1872) was synthesized with ATG at the N‐terminus and a 6×His tag sequence at the C‐terminus. Synthetic cDNA was amplified by PCR using the following primer pair:5′‐CCGAAGCTTATGTGTTGCCAACTCA‐3′ and 5′‐CGGGGATCCTCAGTGATGGTGATGGTGATGAACTCCCAAAG‐3′.

The PCR products were digested with restriction enzymes (Takara Bio Inc.), and the restriction sites (*HindIII* and *BamHI*) are underlined. Then, the genes were ligated into the pFastBac‐Dual expression vector (Invitrogen Inc.), and the fusion sequences were confirmed by sequencing. After that, the fusion pFastBac‐Dual vectors were transformed into bacterial *DH10* cells, and the extracted bacmids were then transfected into Sf9 cells for 4 days to obtain passage 1 baculoviruses (P1 baculoviruses).^10^

### 2.2 AFP and AFP fragment expression in a Bac‐to‐Bac baculovirus system

AFP and AFP fragments were expressed using the Bac‐to‐Bac Baculovirus Expression System (Invitrogen Inc.). The process was as follows: (1) Sf9 insect cells were cultured in SFX‐Insect (Cytiva Ltd.), SF900 II and Grace's medium (Thermo Fisher Ltd.) containing 10% foetal bovine serum (FBS) or without serum at a density of 1 × 10^6^ cells/ml. (2) P1 baculoviruses were amplified to obtain P2 baculoviruses. The amplification process was as follows: 10 ml of P1 baculovirus was added to 100 ml of Sf9 cells cultured in SFX‐Insect, SF900 II and Grace's medium and harvested at 72 h after infection. (3) Fifty milliliters of P2 baculovirus was added to 1 L of Sf9 cells, and secreted AFP and AFP fragments were harvested in the medium at 72 h after infection, as described previously.^10^

### 2.3 Analysis of the expression of secreted AFP and AFP fragments

The insect medium containing secreted AFP and AFP fragments was collected and centrifuged at 4700 *g* for 5 min. Then, 200 μl of 10× HBS buffer (10 mM HEPES [pH 7.2] and 150 mM NaCl) and 100 μl of nickel‐charged resin (Ni‐NTA Agarose; Qiagen Ltd.) were added to 4 ml of supernatant. After the sample was mixed and shaken for 3 h at 4°C, it was centrifuged at 1200 *g* for 2 min and eluted with 200 μl of 300 mM imidazole in HBS buffer. The eluted AFP and AFP fragments were analysed by SDS/PAGE. The reduced protein (denatured) SDS/PAGE sample contained a reducing buffer and was boiled for 10 min. The nonreduced protein sample did not contain a reducing buffer and was not heated.^10^

### 2.4 Expression and purification of the AFP ^390–609^ fragment in a Bac‐to‐Bac baculovirus system

Fifty millilitres of P2 baculoviruses with the AFP^390–609^ fragment gene was added to 1 L of Sf9 cells in SFX‐Insect medium and harvested at 72 h after infection. Then, the medium was centrifuged at 4700 *g* for 5 min, and the supernatant was collected, filtered and concentrated to 100 ml with HBS buffer (10 mM Hepes [pH 7.2] and 150 mM NaCl) by cross‐flow filtration (Millipore Corp.). The concentration was passed through nickel‐charged resin (Ni‐NTA Agarose; Qiagen Ltd.). The AFP^390–609^ fragment was captured by the resin and eluted with 500 mM imidazole. The eluted buffer was concentrated to 1 ml and then further purified by gel filtration chromatography with HBS bufferusing a Superdex 200 Increase 10/300 column (Cytiva Ltd.).^10^ After affinity chromatography, it was centrifuged at 4700 *g* for 1 h by centrifugal filter units (Millipore Corp.) to obtain about concentration of 1 mg/ml protein.

### 2.5 Western blotting

Western blotting was used for analysis of the expression of AFP and AFP fragments in a Bac‐to‐Bac system. The anti‐AFP third domain antibodies used in this experiment were purchased from Beyotime Biotechnology. In addition, the anti‐His tag antibodies were purchased from Sangon Biotech.

### 2.6 Laser confocal microscopy observation AFP ^390–609^ fragment uptake by HCC cells and normal hepatic cells

Firstly, cells were diluted with DMEM (Dulbecco's Modified Eagle Medium) without serum into a suspension of 2 × 10^4^ cells/ml, and 500 μl suspension cells were added into a chamber slide. Then add 30 μl FITC‐AFP^390–609^ fragment (FITC‐AFP^390–609^ fragment link used HOOKDye Labeling Kit). Samples were culture at 37°C avoid light, with 5% CO_2_ for 37 h. Secondly, the cells were washed three times (5 min each) with PBS, added 500 μl 4% paraformaldehyde‐fixed cell 30 min and used PBS wash three times. Thirdly, added 200 μl DAPI for 10 min, wash with PBS for three times, then, added 50 μl fluorescent quencher and observed with an Olympus FluoView FV1000 laser.

### 2.7 MTT assay

Bel7402 cells were plated into 96‐well plates and added with sorafenib or sorafenib + AFP fragments then, cultured in DMEM medium supplemented with 10% FCS at 37°C in a humidified atmosphere of 5% CO_2_ for 72 h. The viability of the cells was measured by methylthiazolyldiphenyl‐tetrazolium bromide (MTT) assay as described. The cells viability = treated group A490/control group A490 × 100%.

### 2.8 RNA interference

Bel7402 cells were cultured until they reached 50% confluence. Then, they were transfected of siRNA‐AFP Lentivirus (HitransG A; Shanghai Genechem Co., Ltd.) The cell lines Bel7402‐siRNA‐AFP which were interfered AFP expression by siRNA were screened by puromycin (Beyotime Biotechnology) after 72 h. The siRNA sequence is as follows: 5′‐ccCTCTTGAATGCCAAGATAA‐3′, use vector GV493 (http://www.genechem.com.cn/service/index.php?ac=gene&at=vector_search&keyword=GV493).^11^

### 2.9 Scratch test

Cell motility was analysed by a scratch repair assay. One day before scratching, Bel7402 cells were treated with sorafenib (5 μg/ml); sorafenib (5 μg/ml) + AFP fragment (AFP^360–609^); and Bel7402‐siRNA‐AFPcells were treated with sorafenib (5 μg/ml), and were seeded into 6‐well plates. A scratching wound was created by scraping the middle of the cell monolayer with a sterile micropipette tip. After all detached cells were washed away with PBS, the cells were cultured with medium containing 10% FBS, and images of cell migration into the wound area were captured at 0 and 72 h by an inverted microscope (100×) to record the distance migrated.^11^

### 2.10 Cell migration was detected by the transwell method

To measure cell migration, transwell chambers were used to observe cultured cell inserts (Transwell chamber; 8 mm pore size; Costar, Corning Co., Ltd). Bel7402 cells (5 × 10^4^) were added to the upper chambers and cultured with serum‐free DMEM and treated with sorafenib (5 μg/ml); sorafenib (5 μg/ml) + AFP fragment (AFP^360–609^); and Bel7402‐siRNA‐AFP cells were treated with sorafenib (5 μg/ml). In addition, the lower chamber was filled with complete medium containing 20% FBS. After 72 h of incubation, the cells in the upper chamber were carefully removed with a cotton swab, and those that had migrated through the membrane to the lower surface were fixed with 90% methanol and stained with 0.1% crystal violet. The number of cells that had migrated through the pores was quantified by counting five in dependent visual fields using a microscope (Olympus) with 20× objective. The migratory cell ratio = numbers of treated groups/numbers of untreated groups × 100%.^11^

### 2.11 Flow cytometry analysis

Bel7402, L‐02 and PLC/PLF/5 cells were cultured in DMEM supplemented with 10% FBS at 37°C in a humidified atmosphere containing 5% CO_2_. Then, the cells were treated with sorafenib (10 μg/ml), sorafenib (10 μg/ml) + siRNA‐AFP or sorafenib (10 μg/ml) + AFP fragment (AFP^360–609^) for 24 h; harvested by trypsinization; washed with PBS; and resuspended and incubated in PE Annexin V solution (BD Biosciences) for apoptosis analysis. At least 20,000 live cells were analysed on a NovoCyte™ flow cytometer. Data were analysed using NovoExpress software.^11^

### 2.12 Statistical analysis

The results of multiple observations were presented as the mean ± SD of three independent experiments. Statistical analysis was determined using one‐way anova and *t*‐test. **p < 0.01 and ***p < 0.001 were statistical significance.

## 3 RESULTS

### 3.1 Analysis of expression vectors

**FIGURE 1:**
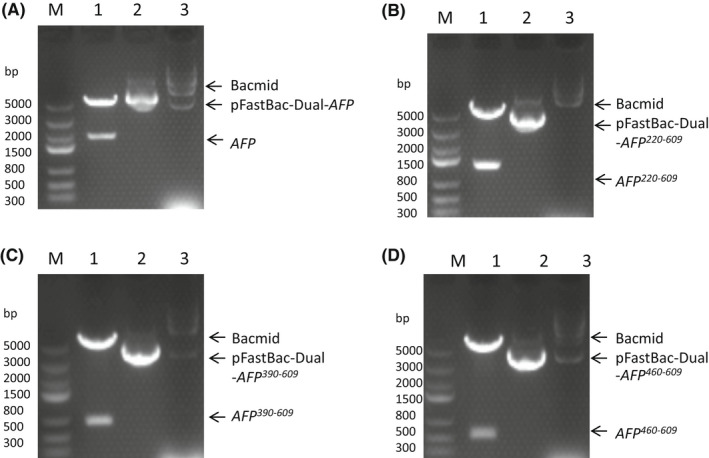
Agarose gel electrophoresis analysis of the recombinant vectors containing the human *afp* and fragment genes. (A) pFastBac‐Dual‐AFP (full‐length AFP) vector, (B) pFastBacDul‐AFP^220–609^ (AFP fragment including residues 220–609 with 6×His) vector, (C) pFastBac‐Dual‐AFP^390–609^ (AFP domain3 fragment including residues 390–609 with 6×His) vector and (D) pFastBac‐Dual‐AFP^460–609^ (AFP domain3 fragment including residues 460–609 with 6×His) vector. Lane M, DNA marker; Lane 1, pFastBac‐Dual‐AFP and fragment vector digested by *BamHI* and *HindIII* restriction enzymes; Lane 2, pFastBac‐Dual‐AFP and fragment vector; Lane 3, recombinant Bacmid.

Figure 1A–D shows the four expression vectors, namely AFP, AFP^220–609^, AFP^390–609^ and AFP^460–609^. Figure 1A–D Lane M shows the DNA marker, and Lane 1 shows that the vectors were digested with *BamHI* and *Hind III* restriction enzymes to release a band measuring approximately 2000 bp (A), 1200 bp (B), 660 bp (C) and 450 bp (D), confirming the presence of the recombinant human *afp* gene and the human *afp* fragment genes. DNA sequencing of the four recombinant vectors also confirmed the correct gene sequences. Figure 1A–D Lane 2 shows the recombinant vectors, and Lane 3 shows the correct homologous recombination that occurred in the bacmid.

### 3.2 Analysis of AFP and fragments expression with 10% FBS or without serum

**FIGURE 2:**
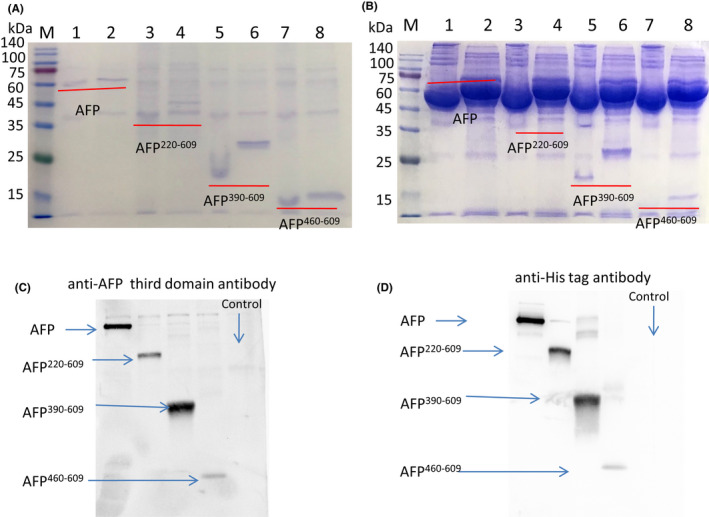
AFP and fragments expression in SFX‐Insect medium. P1 virus‐infected Sf9 cells were cultured in SFX‐Insect medium for 72 h, and the medium was collected and analysed by SDS‐PAGE and Western blotting. (A) Medium without serum. Lanes 1 and 2: AFP (Lanes 1 nonreduced or native AFP, Lanes 2 reduced rAFP); Lanes 3 and 4: AFP^220–609^ (Lanes 3 nonreduced or native AFP^220–609^, Lanes 4 reduced AFP^220–609^); Lanes 5 and 6 AFP^390–609^ (Lanes 5 nonreduced or native AFP^390–609^, Lanes 6 reduced AFP^390–609^); Lanes 7 and 8 AFP^460–609^ (Lanes 5 nonreduced or native AFP^460–609^, Lanes 6 reduced AFP^460–609^). (B) SFX‐Insect medium with 10%FBS (foetal bovine serum). Lanes 1 and 2: AFP, Lanes 3 and 4: AFP^220–609^, Lanes 5 and 6: AFP^390–609^, Lanes 7 and 8: AFP^460–609^ fragment. (C) Western blotting with anti‐AFP third domain antibody and (D) Western blotting with anti‐His tag antibody. Control: medium collected from un‐transfected cells.

Sf9 cells cultured in SFX‐Insect medium were transfected with P1 virus, and secreted recombinant AFP and fragments (the expressed protein secreted into the insect medium) were assessed by SDS/PAGE. The results showed that all four recombinant secreted proteins could be expressed and detected in the medium (Figure 2A: 1–8). The reduced AFP molecular mass was approximately 70 kDa, the AFP^220–609^ fragment molecular mass was approximately 43 kDa, the AFP^390–609^ fragment molecular mass was approximately 26 kDa, and the AFP^460–609^ fragment molecular mass was approximately 16 kDa, consistent with the sizes of the *afp* gene and the *afp* fragment gene. The nonreduced or native proteins were slightly lower than the reduced proteins in the SDS/PAGE gel because they were nonlinear and ran a little fast.

To test whether FBS (foetal bovine serum) could increase the expression level, 10% FBS was added to the medium, and it was found that 10% FBS did not increase the expression level (Figure 2B: 1–8); moreover, FBS affected the purification of protein.

To further confirm the bands are representing the corresponding AFP fragments, Western blotting was performed with anti‐AFP third domain antibody and with anti‐His tag antibody. The result showed the expressed proteins were correct (Figure 2C,D).

### 3.3 Analysis of fragment expression in different media

**FIGURE 3:**
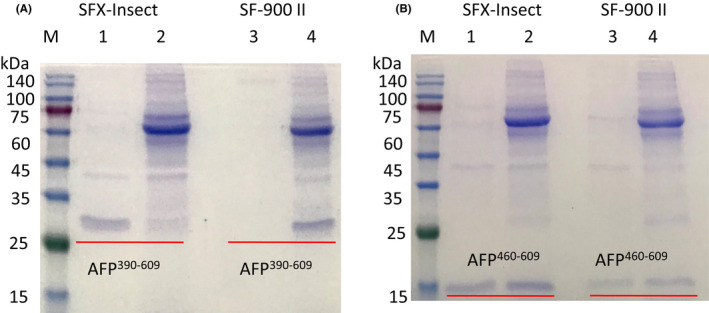
AFP^390–609^ and AFP^460–609^ fragments expressed in different media. (A) AFP^390–609^ fragment expressed in SFX‐Insect and SF900 II media, Lanes 1 and 2: SFX‐Insect medium, Lanes 3 and 4: SF900 II medium; Lanes 1 and 3: without FBS; Lanes 2 and 4: FBS concentration of 2%. (B) AFP^460–609^ fragment expressed in SFX‐Insect and SF900 II media. Lanes 1 and 2: SFX‐Insect medium. Lanes 3 and 4: SF900 II medium. Lane 1–3: without FBS; Lane 2 and Lane 4: FBS concentration of 2%.

To test different medium expression levels, P1 viruses with AFP^390–609^ and AFP^460–609^ fragments were transfected into Sf9 cells cultured in different media with or without 2% FBS. The secreted recombinant fragments extracted from the medium were assessed by SDS/PAGE, and the results showed that the AFP^390–609^ fragment could be expressed in SFX‐Insect medium with or without 2% FBS. However, the fragment could not be expressed in SF900 II medium without FBS (Figure 3A). The AFP^460–609^ fragment could be expressed in SFX‐Insect and SF900 II medium with or without 2% FBS, but the expression in SFX‐Insect medium was higher than that in the SF900 II medium (Figure 3A,B). Grace's medium did not express the fragments (data not shown). Therefore, it is better to express the AFP recombinant fragments in SFX‐Insect medium.

### 3.4 Purification of the AFP ^390–609^ fragment

**FIGURE 4:**
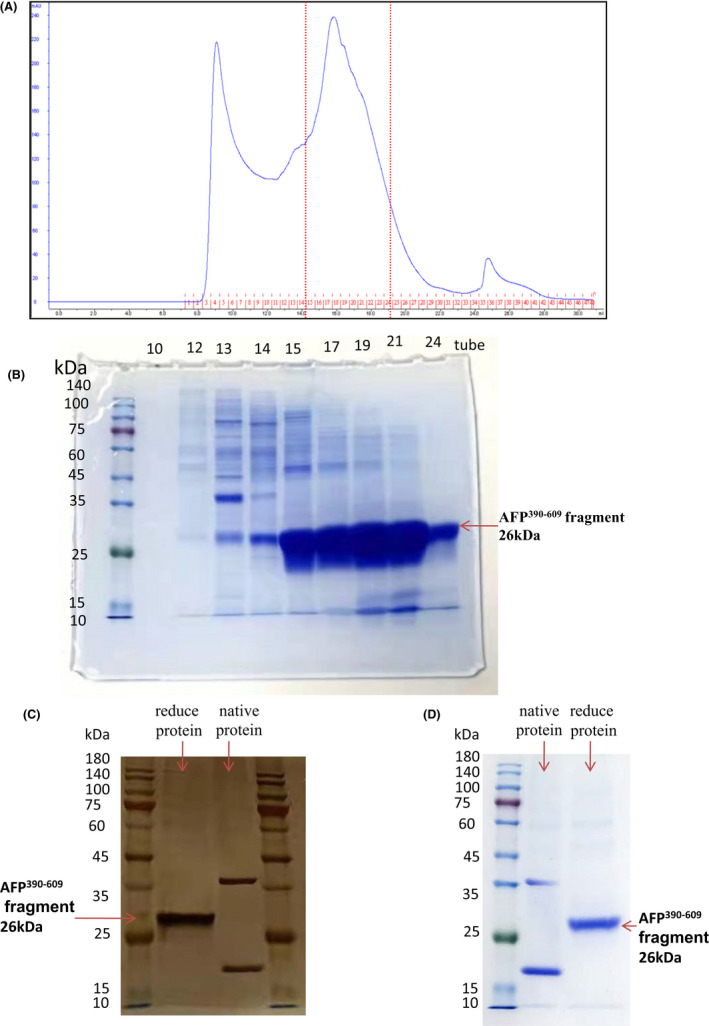
Analysis of the purified AFP^390–609^ fragment. (A) Gel filtration chromatography of the AFP^390–609^ fragment purification. (B) Coomassie‐blue staining analysis of the AFP^390–609^ fragment eluted from Gel filtration chromatography (No.10 tube to No.24 tube fractions). (C) silver staining (the amount of protein loaded in each lane was 10 μl). (D) Coomassie‐blue staining analysis of purified the AFP^390–609^ fragment.

It was found that the AFP^390–609^ fragment was highly expressed, so we further expressed a certain amount and purified the protein in the system. Figure 4A shows the purification chromatography of the secreted AFP^390–609^ fragment from 1 L of medium. The peak value was approximately 240 mAU, which indicated that the purification process was successful and that a certain amount of the fragment protein was obtained. The Eluted fractions (from No.10 tube to No.24 tube) were collected and analysed by Coomassie‐blue staining (Figure 4B); it was found that No.15–No.24 tube contained high concentration of AFP^390–609^ fragment. The fragment protein in No.15–No.24 tube was collected and further purified by gel filtration chromatography. The last purified AFP^390–609^ fragment was analysed by silver staining and Coomassie‐blue staining (Figure 4C,D). The molecular mass was approximately 26 kDa, which is consistent with the size of the *afp* gene fragment. The native protein formed two bands, the high band may be dimers, and the low band was monomer which ran slightly lower than the reduced proteins in the SDS/PAGE gel because they were nonlinear and ran a little fast.

The AFP^390–609^ protein was further analysed by LC/LC–MS/MS^12^ and the results showed that the molecular weight of the purified sample was 25,834 Da. In total, 88% of the protein sequence was determined and matched the sequence of AFP^390–609^ (Figure S1), indicating that AFP^390–609^ was correctly expressed and purified. The purified AFP^390–609^ fragment is soluble and its isoelectric point (IP) at pH 5.86.

### 3.5 The AFP ^390–609^ fragment (named AFP fragment briefly) uptake by Bel7402 cells

**FIGURE 5:**
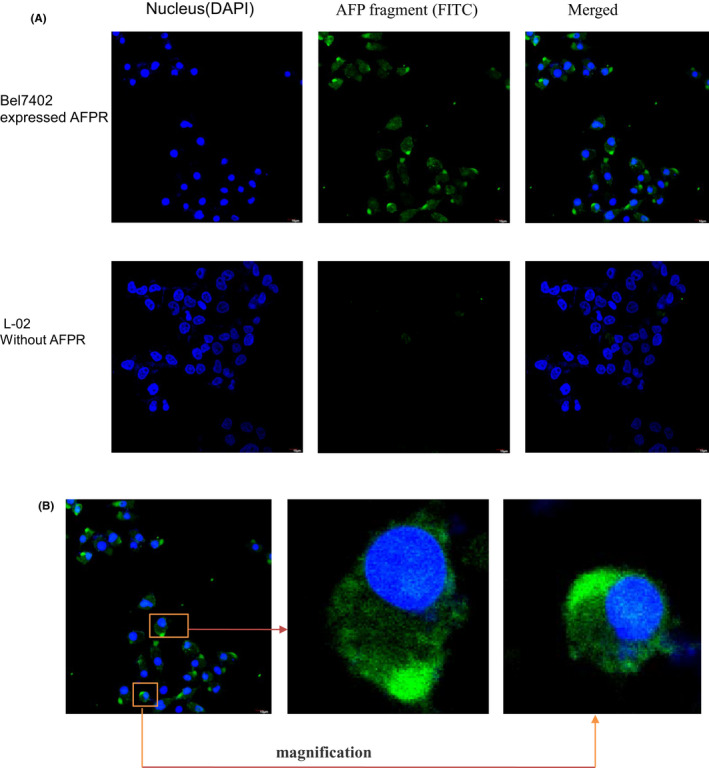
Localization of AFP^390–609^ fragment (or named AFP fragment) in Bel7402 and L‐02 cells. (A) Bel7402 and L‐02 cells were added with AFP fragment, followed by culturing at 37°C in a humidified atmosphere of 5% CO_2_. The images were captured under laser confocal microscopy. Localization of AFP fragment was visualized in Bel7402 (HCC cells with high AFP receptor expressed) but not in L‐02 cells (normal hepatic cell). (B) Localization of AFP fragment in Bel7402 was magnification. Nuclei are stained with DAPI (blue). AFP fragment was labelled with FITC (green). Three independent experiments were performed for these data.

To test the AFP^390–609^ fragment uptake by HCC cells with high AFP receptor expressed, immunofluorescence and confocal microscope were observed using Bel7402 cells as model (HCC cells which highly expressed AFP receptor). Figure 5A showed that AFP^390–609^ fragment was uptake by Bel7402 cells, but it was not uptake by the L‐02 cells (normal hepatic cells which did not expressed AFP receptor). It was also found that the uptake AFP^390–609^ fragment was mainly concentrated in the cell bulge that might affect the communication and growth between cells (Figure 5B).

### 3.6 AFP ^390–609^ fragment synergize with sorafenib to inhibit the growth of Bel7402

**FIGURE 6:**
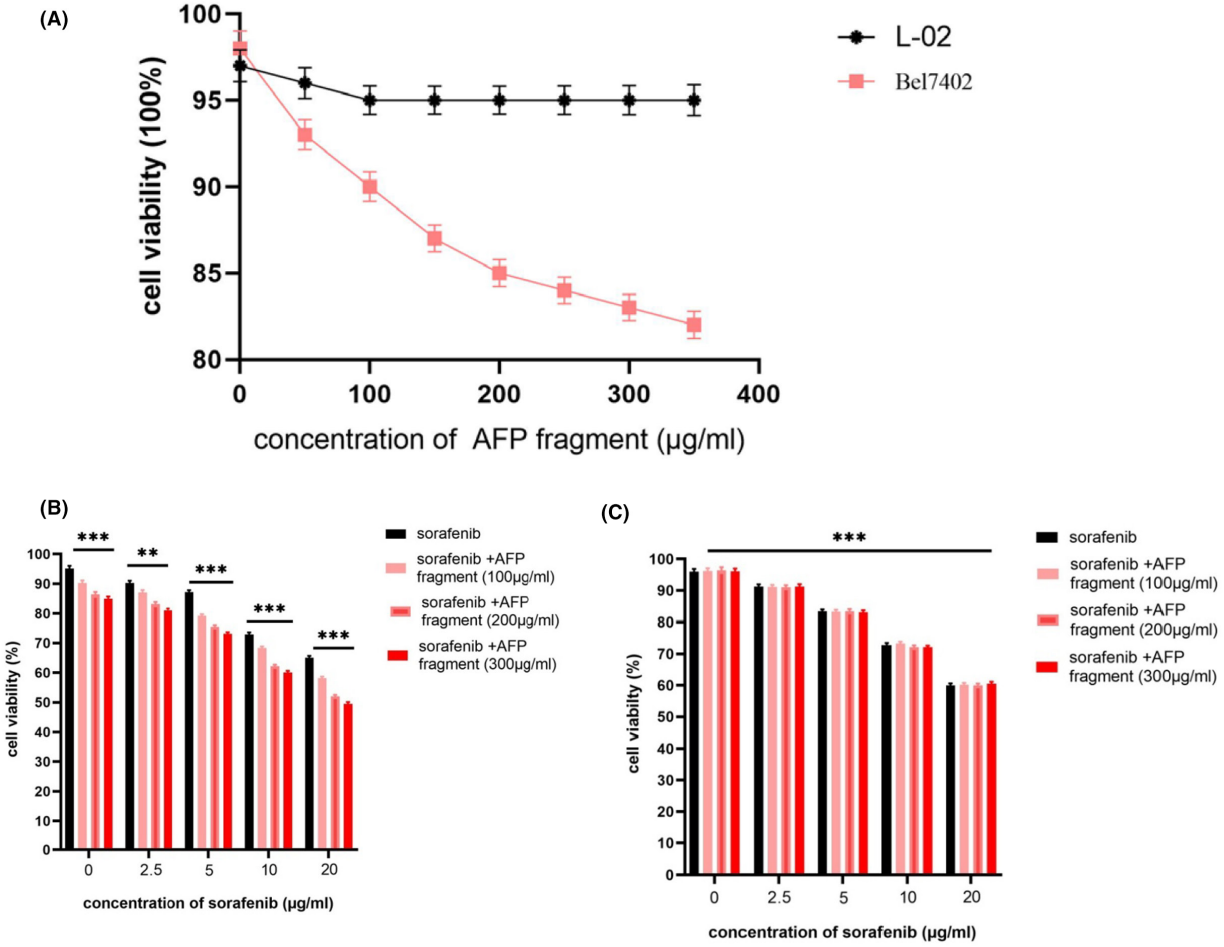
Effects of and AFP^390–609^ (AFP fragment) and sorafenib on the growth of human hepatoma cells Bel7402 and human normal liver cells L‐02. (A) The human hepatoma cell line Bel7402 and normal hepatic cell L‐02 were treated with AFP fragment at concentrations of 0–350 μg/ml. The viability of the cells was analysed by MTT. (B) Bel7402 and (C) L‐02 cells were treated with sorafenib at concentrations of 2.5–20 μg/ml; at the same time synergized with AFP fragment at concentrations of 100, 200 and 300 μg/ml for 72 h. The viability of the cells was analysed by MTT. **p < 0.01 and ***p < 0.001 versus untreated groups. *N* = 3.

To test AFP^390–609^ fragment bioactivity, MTT assay was conducted. The results indicated that AFP^390–609^ fragment suppressed Bel7402 cell growth. The viability of Bel7402 cells decreased to 85% when treated with 200 μg/ml AFP^390–609^ fragment. However, the fragment did not appreciably affect normal L‐02 cell growth, and the viability of L‐02 cells decreased slightly to approximately 5% when exposed to corresponding concentration (Figure 6A).

To detect the AFP^390–609^ fragment in effect the anticancer drug activity, it affect sorafenib was analysis by MTT. The results indicated that Bel7402 and L‐02 treated with different concentrations of sorafenib (2.5–20 μg/ml), the growth (viability) of these cells was significantly inhibited when sorafenib was present at ≥10 μg/ml, and the viability of Bel7402 cells was significantly suppressed synergizes with AFP^390–609^ fragment (Figure 6B), but L‐02 cells were not significantly suppressed synergizes with AFP^390–609^ fragment (Figure 6C). This result indicated that AFP^390–609^ fragment was able to synergize with sorafenib to inhibit growth of Bel7402 which highly expressed AFP receptor, and when the AFP fragment concentration increased to 300 μg/ml, the synergized effect with sorafenib was similar to 200 μg/ml. So, we chose 200 μg/ml of AFP fragment in the follow functional assays.

### 3.7 AFP ^390–609^ fragment synergizes with sorafenib to suppress Bel7402 cell scratch repair and migration

To further detect the AFP^390–609^ fragment function in effect the drug activity, the effects of AFP^390–609^ fragment combine with sorafenib on scratch repair and the migration were also observed using Bel7402 cells as model. Because MTT showed that the viability of Bel7402 was significantly inhibited when sorafenib was present at concentration ≥10 μg/ml and not much effect at 5 μg/ml. The high concentration sorafenib (≥10 μg/ml) can cause the cells death and apoptosis that will affect the result in the scratch repair and migration experiment very much. So, we chose 5 μg/ml of sorafenib (the concentration does not much affect at the cell viability) and extended incubation period for 72 h in the scratch repair and migration experiments.

Scratch repair assays indicated that the control cells covered more than 85% scratch area. However, when Bel7402 cells were treated with sorafenib (5 μg/ml) for 72 h, the cells covered 45% of the scratch area, when Bel7402 cells were treated with sorafenib (5 μg/ml) + AFP fragment (AFP^390–609^), the cells covered only 30%.

**FIGURE 7:**
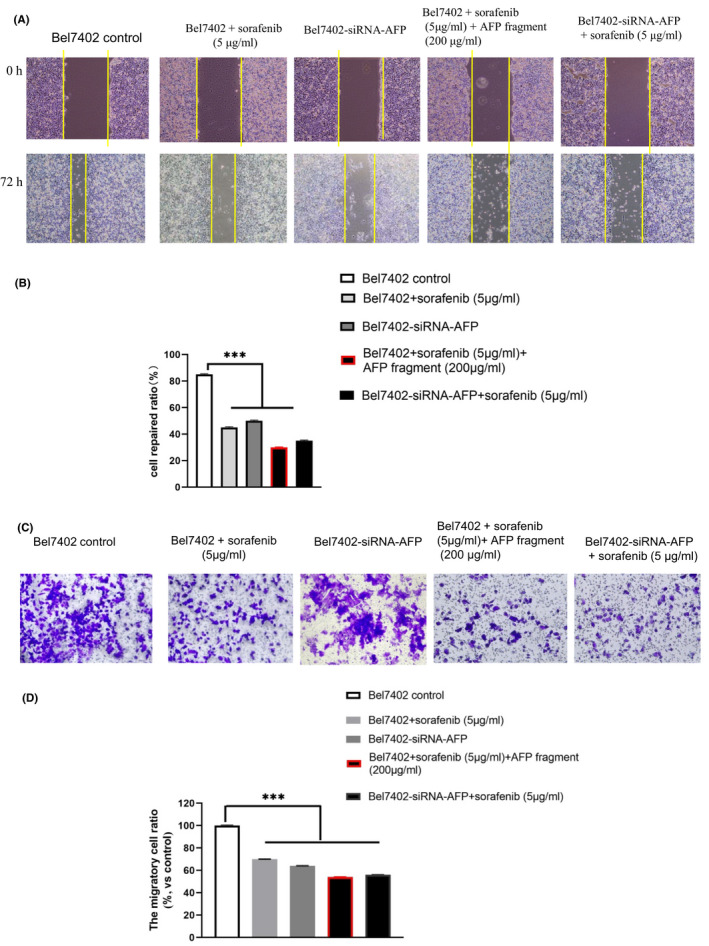
AFP^390–609^ fragment synergizes with sorafenib to inhibit scratch repair and migration of Bel7402 cells. (A) Bel7402 cells were treated with sorafenib (5 μg/ml); sorafenib (5 μg/ml) + AP fragment (200 μg/ml); or Bel7402‐siRNA‐AFP cells were treated with sorafenib (5 μg/ml) for 72 h; and the scratch area of Bel7402 cells covered was detected in scratch repair experiment. (B) The column picture indicates the statistical analysis of the cell repair ratio. (C) The migration of Bel7402 cells and Bel7402‐siRNA‐AFP cells (treated as A) was detected in a transwell chamber. (D) The column picture indicates the statistical analysis of the migratory cell ratio. ***p < 0.001 versus control groups. The images are representative of at least three independent experiments.

The Bel7402‐siRNA‐AFP cells which were silenced AFP expression by siRNA (Figure S2) covered about 50% scratch area, when Bel 7402‐siRNA‐AFP cells treated with sorafenib (5 μg/ml), the cells covered 35% scratch area (Figure 7A,B). These results indicated that AFP^390–609^ fragment and siRNA‐AFP harbour a function to synergize with sorafenib to suppress Bel7402 cells scratch repair.

The migration assays shown the similar result. When Bel7402 cells were treated with sorafenib (5 μg/ml) for 72 h, the migration ratio of the cells was 70% compared with the control group, when Bel7402 cells were treated with sorafenib (5 μg/ml) + AFP fragment (AFP^390–609^), the migration ratio of the cells was 54%. In addition, the migration ratio of Bel7402‐siRNA‐AFP cells was 64%, when Bel7402‐siRNA‐AFP cells treated with sorafenib (5 μg/ml), the migration ratio of the cells was 56% (Figure 7C,D).

These results demonstrated that the AFP^390–609^ fragment could also synergize with sorafenib to inhibit migration in Bel7402 cells. The effect of AFP^390–609^ on migration and scratch repair of Bel7402 cells were similar to that of siRNA‐AFP, suggesting that AFP^390–609^ may inhibit the Bel7402 cells (HCC with highly AFP receptor expressed) migration by regulating the AFP signalling pathway.

### 3.8 AFP ^390–609^ fragment synergizes with sorafenib to promote apoptosis of Bel7402 cells

To further detect the bioactivity of the AFP^390–609^ fragment in the present study, we also selected Bel7402 cells to examine the effects of AFP^390–609^ on cellular apoptosis synergized with sorafenib. In the apoptosis experiment, it was found that cell apoptosis not much affected by 5 μg/ml of sorafenib (data not shown). So, it chose treatments 10 μg/ml of sorafenib for 24 h that could get statistical significance of result.

Flow cytometric analysis demonstrated that when Bel7402 cells were treated with sorafenib (10 μg/ml) for 24 h, the apoptosis ratio of the cells was 30%, and after treatment with sorafenib (10 μg/ml) + AFP^390–609^ fragment (200 μg/ml), the apoptosis ratio of the cells was increased to 45%.

**FIGURE 8:**
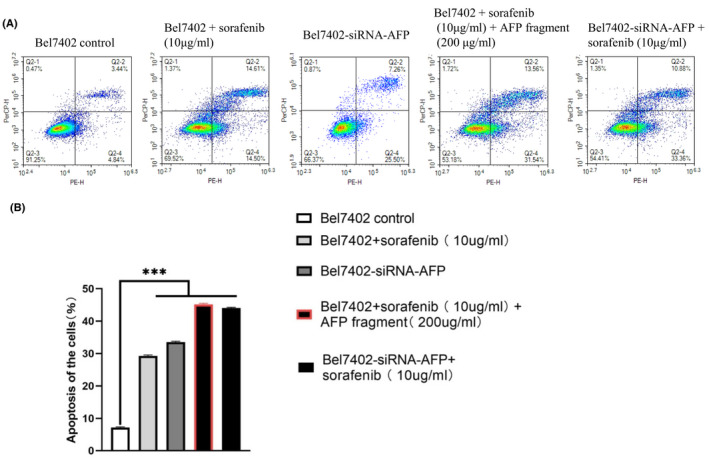
AFP^390–609^ fragment synergizes with sorafenib to promote the apoptosis of Bel7402 cells. (A) Bel7402 cells were treated with sorafenib (10 μg/ml), sorafenib (10 μg/ml) + AFP fragment (200 μg/ml) or Bel7402‐siRNA‐AFP cells treated with sorafenib (10 μg/ml) for 24 h; the apoptosis of Bel7402 cells was analysed by flow cytometry. (B) The column picture shows the statistical analysis of the apoptosis ratios; ***p < 0.001 versus control groups. The images are representative of at least three independent experiments.

The apoptosis ratio of Bel7402‐siRNA‐AFP cells was 33%, when Bel7402‐siRNA‐AFP cells treatment with sorafenib (10 μg/ml) the apoptosis ratio of the cells was 44%. These results demonstrated that the AFP^390–609^ fragment also synergized with sorafenib to promote apoptosis in Bel7402 cells (Figure 8A,B), and the effect of AFP^390–609^ on hepatoma cell apoptosis was similar to that of siRNA‐AFP, also suggesting that the AFP^390–609^ fragment may promote apoptosis by inhibiting AFP signalling.

## 4 DISCUSSION

Alpha fetoprotein is a crucial molecule that promotes proliferation or drug resistance in liver cancer cells. AFP promotes the growth of human HCC by antagonizing paclitaxel and suppressing the expression of activated caspase‐3. AFP is involved in multidrug resistance (MDR) by activating the PI3K/AKT/mTOR signalling pathway, which leads to metabolic reprogramming of cancer stem cells, inhibition of the expression of apoptosis‐related enzymes and resistance to tumour cell apoptosis.^13^, ^14^, ^15^

Alpha fetoprotein domain3 fragments can be used to inhibit AFP malignancy by completing AFP to interact with signalling molecules and affect cancer cell growth, drug resistance and metastasis. For example, rAFP3D can recognize and bind to the AFPR of cancer cells, which can increase the intake and enhance the accumulation of drugs in cancer cells, and also strengthen the cytotoxic effect of drugs. rAFP3D delivery of doxorubicin (Dox) can increase the sensitivity of human ovarian carcinoma cells and breast cancer cells to Dox.^8^ Some recombinant fragment proteins of AFP domain3 have been reported, such as rAFP3D, which has residues 404–609^8^; r3dAFP, which has residues 357–590^7^; and AFP‐3 BC, which has residues 473–596,^6^ these fragments can deliver drugs to destroy cancer cells.

Here, we report a new AFP domain3 fragment containing residues 390–609 with 6×His that was expressed in a Bac‐to‐Bac system. The bioactivity of the fragment was detected in the HCC cell line Bel7402. These results indicated that the AFP fragment can synergize with sorafenib to inhibit the hepatoma cell growth and migration and promote the apoptosis.

Sorafenib is a multitarget drug for the treatment of inoperable HCC or distant metastasis. It has been one of the good choices for the first‐line treatment of advanced HCC for decades. However, sorafenib can only extend the average life expectancy of patients by approximately 1 year, and drug resistance is usually acquired within 6 months, suggesting that HCC easily acquires resistance to sorafenib.^16^, ^17^ Drug resistance to sorafenib remains incompletely elucidated. AFP may neutralize sorafenib‐induced death signals and abnormalities in signalling proteins such as AKT, VEGF and EGFR,^18^ promotes cell growth and inhibit tumour cell apoptosis.^19^ High expression of AFP in HCC could induce drug resistance result in sorafenib treatment failure. In the 2018 EASL (European Association for the Study of the Liver)^20^ clinical practice guidelines, it suggests that AFP can be used as an indicator for the diagnosis and prognosis of advanced HCC. AFP can activate the PI3K/AKT signalling, stimulates the transcription cofactor mTOR, STAT3, HIF‐1α and Bcl‐2, which regulates the expression of oncogenes, as well as promotes angiogenesis and the growth of the hepatoma cells. Many HCC patients with elevated AFP expression may have drug resistance and a poor prognosis.^4^, ^21^ Therefore, it is urgent to find a way to inhibit AFP malignancy in treatment of HCC.

In this report, it was found that a new AFP fragment can synergize with sorafenib to inhibit HCC line Bel7402 growth and migration and promote the apoptosis. The effects of AFP fragments (AFP^390–609^) on Bel7402 apoptosis and migration were similar to those of siRNA‐AFP, suggesting that AFP^390–609^ fragments may inhibit the signalling pathway mediated by AFP. We also found similar results in other HCC cells, such as PLC/PLF/5, which highly expressed AFPR (data not shown). However, the fragment did not have much effect on normal hepatocyte L‐02 cells with low AFPR expression. This indicated that the AFP^390–609^ fragment may compete with AFP to bind to AFPR or be a single molecule to prevent AFP malignant behaviour. The AFP fragment could inhibit PI3K/AKT signalling which induced by AFP,^13^ and sorafenib could block the RAF/MEK/ERK, MAPK and EMT pathway.^22^ These pathways play key roles in the development of HCC. This also indicated that AFP fragment could synergize with sorafenib to block the important pathways that involve in tumour growth, proliferation and metastasis, result in inhibit angiogenesis, and promote apoptosis in HCC. These studies show that the AFP fragment can be a promising vector to deliver drugs or can act directly as a drug to synergize with sorafenib in the treatment of HCC.

## AUTHOR CONTRIBUTIONS

**Bo Lin:** Conceptualization (equal); data curation (equal); formal analysis (equal); funding acquisition (equal); investigation (equal); methodology (equal); project administration (equal). **Qiujiao Wang:** Conceptualization (equal); data curation (equal); methodology (equal). **Wei Li:** Conceptualization (equal); data curation (equal); formal analysis (equal). **Minni Zhang:** Conceptualization (equal); data curation (equal); formal analysis (equal); software (equal); supervision (equal); visualization (equal); writing – original draft (equal). **Zijuan Zou:** Data curation (equal); formal analysis (equal); investigation (equal); methodology (equal). **Xu Dong:** Conceptualization (equal); investigation (equal); validation (equal); visualization (equal). **Yi Chen:** Formal analysis (equal); methodology (equal); project administration (equal). **Junnv Xu:** Investigation (equal); methodology (equal); project administration (equal). **Mingyue Zhu:** Conceptualization (equal); data curation (equal); funding acquisition (equal). **Mengsen Li:** Conceptualization (equal); data curation (equal); formal analysis (equal); funding acquisition (equal); investigation (equal); resources (equal); writing – original draft (equal); writing – review and editing (equal).

## FUNDING INFORMATION

This work was supported by Hainan Province Science and Technology Special Fund (no. ZDYF2021SHFZ222), the National Natural Science Foundation of China (nos. 82060514, 81960519, 81660463, 81560450 and 31560243), the Natural Science Foundation of Hainan Province (nos. 820RC634, 822RC700, 821RC1065, 2019CXTD406, 2019CR204 and 20168263) and the Research Project of Take off the Proclamation and Leadership in Hainan Medical College Natural Science Foundation (no. JBGS202106). Hainan Provincial Association for Science and Technology Program of Youth Science Talent and Academic Innovation (no. QCXM 201922).

## CONFLICT OF INTEREST

The authors declare that this article content has no conflict of interest. All authors of this paper have read and consented to publication of the final version

## Supporting information


Figure S1‐S2
