# Supplementary material for: α‐Fetoprotein fragment synergizes with sorafenib to inhibit hepatoma cell growth and migration and promote the apoptosis
Source: J Cell Mol Med. 2022 Sep 30;26(21):5426–38. doi: 10.1111/jcmm.17565 (PMC9639031; doi:10.1111/jcmm.17565)
Supplement: Supplementary file 1 — Figure S1‐S2 [file JCMM-26-5426-s001.docx]

**Supplementary Figure1. Analysis of purified the AFP^390-609^ fragment** by **LC/LC–MS/MS.**

**A.** The matched peptide sequences. **B.** Mass spectrometry of the representative protein sequence MAATAATCCQLSEDKLLACGEGAADIIIGHLCIR in the AFP^390-609^ fragment.


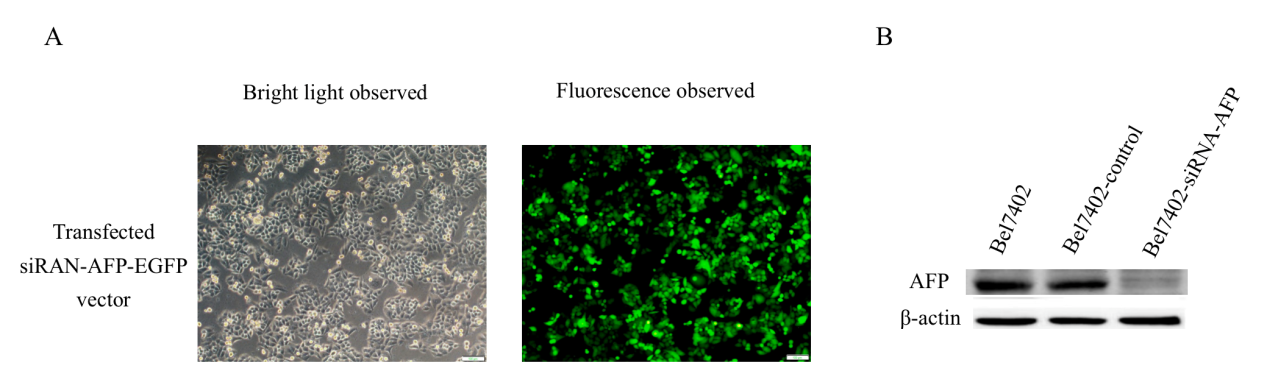


**Supplementary Figure2. Analysis of RNA interference of AFP in Bel7402-siRNA-AFP cells**

**A.** Bright light and Fluorescence microscopy (4×magnification) observation of Bel7402- siRNA-AFP cells which transformed siRNA-AFP-EGFP (enhanced green fluorescent protein). **B.** Westbloting analysis of Bel7402- siRNA-AFP cells which transformed siRNA-AFP.
